# Relevance of Interleukin-6 and D-Dimer for Serious Non-AIDS Morbidity and Death among HIV-Positive Adults on Suppressive Antiretroviral Therapy

**DOI:** 10.1371/journal.pone.0155100

**Published:** 2016-05-12

**Authors:** Birgit Grund, Jason V Baker, Steven G. Deeks, Julian Wolfson, Deborah Wentworth, Alessandro Cozzi-Lepri, Calvin J. Cohen, Andrew Phillips, Jens D. Lundgren, James D. Neaton, INSIGHT SMART/ESPRIT/SILCAAT Study Group

**Affiliations:** 1 School of Statistics, University of Minnesota, Minneapolis, MN, United States of America; 2 Hennepin County Medical Center, Minneapolis, MN, United States of America; 3 Department of Medicine, University of Minnesota, Minneapolis, MN, United States of America; 4 University of California San Francisco, San Francisco, CA, United States of America; 5 San Francisco General Hospital, San Francisco, CA, United States of America; 6 Division of Biostatistics, School of Public Health, University of Minnesota, Minneapolis, MN, United States of America; 7 University College London, London, United Kingdom; 8 Medical Affairs Department, Gilead Sciences, Foster City, CA, United States of America; 9 Department of Infectious Diseases, Rigshospitalet, University of Copenhagen, Copenhagen, Denmark; University of Malaya, MALAYSIA

## Abstract

**Background:**

Despite effective antiretroviral treatment (ART), HIV-positive individuals are at increased risk of serious non-AIDS conditions (cardiovascular, liver and renal disease, and cancers), perhaps due in part to ongoing inflammation and/or coagulation. To estimate the potential risk reduction in serious non-AIDS conditions or death from any cause that might be achieved with treatments that reduce inflammation and/or coagulation, we examined associations of interleukin-6 (IL-6), D-dimer, and high-sensitivity C-reactive protein (hsCRP) levels with serious non-AIDS conditions or death in 3 large cohorts.

**Methods:**

In HIV-positive adults on suppressive ART, associations of IL-6, D-dimer, and hsCRP levels at study entry with serious non-AIDS conditions or death were studied using Cox regression. Hazard ratios (HR) adjusted for age, gender, study, and regression dilution bias (due to within-person biomarker variability) were used to predict risk reductions in serious non-AIDS conditions or death associated with lower “usual” levels of IL-6 and D-dimer.

**Results:**

Over 4.9 years of mean follow-up, 260 of the 3766 participants experienced serious non-AIDS conditions or death. IL-6, D-dimer and hsCRP were each individually associated with risk of serious non-AIDS conditions or death, HR = 1.45 (95% CI: 1.30 to 1.63), 1.28 (95% CI: 1.14 to 1.44), and 1.17 (95% CI: 1.09 to 1.26) per 2x higher biomarker levels, respectively. In joint models, IL-6 and D-dimer were independently associated with serious non-AIDS conditions or death, with consistent results across the 3 cohorts and across serious non-AIDS event types. The association of IL-6 and D-dimer with serious non-AIDS conditions or death was graded and persisted throughout follow-up. For 25% lower “usual” IL-6 and D-dimer levels, the joint biomarker model estimates a 37% reduction (95% CI: 28 to 46%) in the risk of serious non-AIDS conditions or death if the relationship is causal.

**Conclusions:**

Both IL-6 and D-dimer are independently associated with serious non-AIDS conditions or death among HIV-positive adults with suppressed virus. This suggests that treatments that reduce IL-6 and D-dimer levels might substantially decrease morbidity and mortality in patients on suppressive ART. Clinical trials are needed to test this hypothesis.

## Introduction

Among HIV-positive adults with high CD4 cell counts on effective antiretroviral therapy (ART), serious non-AIDS conditions (SNA) are the primary cause of severe morbidity and mortality [1–4]. Among HIV-positive adults, higher levels of interleukin-6 (IL-6), D-dimer, and high-sensitivity C-reactive protein (hsCRP) are associated with an increased risk of cardiovascular disease (CVD) [5–7], cancer [8], and all-cause mortality [9–11]. While ART decreases IL-6, D-dimer and hsCRP levels [10, 12], these biomarkers remain elevated relative to the general population even when the plasma HIV RNA is suppressed [13]. Markers of inflammation and coagulation have been widely studied in the general population, and related to higher risk of CVD [14–22], cancer [23–25], kidney function decline [26] and all-cause mortality [27–29]. These data collectively suggest that chronic inflammation and/or hyper-coagulation contribute to the pathogenesis of these serious non-AIDS events during otherwise effective ART.

Given the assumed, albeit unproven, role of these pathways in causing disease, both vascular and non-vascular, there is intense interest in studying interventions that reduce inflammation and/or coagulation [30, 31]. To date, no single biomarker has been formally validated as a surrogate marker for non-AIDS morbidity. We developed a biomarker score (the “IL-6 & D-dimer score”), which optimally combines markers of inflammation and coagulation to predict the risk of serious non-AIDS conditions or death from any cause (SNA/death) among HIV-infected adults on suppressive ART, and a biomarker model to estimate the effect of sustained long-term reductions in IL-6 and D-dimer levels on the risk of SNA/death. The IL-6 & D-dimer score could be a suitable biomarker endpoint for phase II trials aiming to compare treatments side-by-side for their potential to reduce serious non-AIDS conditions, even if the treatments have different mechanisms of action, or target inflammation and/or coagulation to varying degrees.

## Methods

### Population

Our analysis includes 3766 HIV-positive participants on suppressive ART from the control arms of three studies performed by the INSIGHT trials network: the Strategies for Management of Antiretroviral Therapy (SMART; 1748 participants) [32, 33], Evaluation of Subcutaneous Proleukin® in a Randomized International Trial (ESPRIT; 1446 participants) and Subcutaneous Recombinant, Human Interleukin-2 in HIV-Infected Patients with Low CD4+ Counts under Active Antiretroviral Therapy (SILCAAT; 572 participants) [34] trials. All participants were using ART at study entry, had HIV RNA levels ≤ 500 copies/mL, had been randomized to continuing suppressive ART, and provided consent to store blood for future research. We restricted our analysis to participants who were treated and virally suppressed, because early treatment and viral suppression is increasingly the norm, and because effective ART also decreases levels of inflammation and coagulation [10, 12]. These three studies comprised all large HIV trials conducted by the INSIGHT network in the past 15 years that enrolled ART-experienced participants.

The study was conducted in accordance with the declaration of Helsinki. The SMART, ESPRIT and SILCAAT trials were approved by the Institutional Review Board at the University of Minnesota and the institutional review boards or institutional ethics committees at each of the participating clinical sites worldwide. The Institutional Review Board at the University of Minnesota also approved plans for the analysis of stored specimens for consenting participants. All participants provided written informed consent. Clinicaltrials.gov identifiers: NCT00027352, NCT00004978 and NCT00013611.

### Outcome measures

The primary outcome was SNA/death, a composite endpoint comprising cardiovascular disease (myocardial infarction, stroke, or death due to cardiovascular disease including sudden death not attributed to other causes), end-stage renal disease, decompensated liver cirrhosis, non-AIDS cancer (excluding non-fatal non-melanoma skin cancer), and death due to any cause. In the SMART and ESPRIT trials, clinical events were assessed by an independent Endpoint Review Committee (ERC), blinded to randomization assignment. Only outcomes assessed as confirmed or probable were included. In the SILCAAT study, SNA events were identified from serious adverse event reports. Non-fatal events were coded according to the Medical Dictionary for Regulatory Activities (MedDRA®) version 12.0 [35], causes of death according to the CoDe system [36].

### Biomarkers

We focused on IL-6, hsCRP, and D-dimer for several reasons. First, in a panel of 6 plasma biomarkers measured in the INSIGHT SMART study, these 3 biomarkers were strongly associated with all-cause mortality, which motivated us to determine these markers also for the ESPRIT and SILCAAT trials, using stored baseline plasma specimens.[6] Second, higher levels of IL-6, hsCRP and D-dimer have been associated with increased risk of CVD, cancer, and all-cause mortality among people living with HIV [5–11], as well as in the general population [14–25, 27–29]. Third, the biomarkers have high laboratory and biological reproducibility. [37]

Levels of IL-6, D-dimer and hsCRP at study entry were measured on plasma collected at baseline for all patients who provided written consent, and stored at -70 degrees Celsius. For the SMART study, the biomarkers were determined centrally at the Laboratory for Clinical Biochemistry Research at the University of Vermont (Burlington), for the ESPRIT and SILCAAT trials, by SAIC-Frederick (Frederick, MD). IL-6 was measured by ELISA assays by R&D Systems, Minneapolis, MN, using the QuantiGlo® Chemiluminescent ELISA (range 0.48–1500 pg/mL) for SMART and the high-sensitivity Quantikine® HS ELISA (range 0.16–10 pg/mL) for ESPRIT and SILCAAT samples. The QuantiGlo® Chemiluminescent ELISA was chosen for SMART because HIV-positive populations have higher levels of IL-6 than the general population, and this assay’s range is wider while still giving sufficient precision at the lower limit. Our laboratories validated that the two R&D ELISA assays provided similar measurements, using split samples. D-dimer was measured by ELISA on the Sta-R analyzer, Liatest D-DI (Diagnostic Stago, Parsippany, NJ, USA) for the SMART study, and on a VIDAS instrument (BioMerieux Inc., Durham, NC, USA) for ESPRIT and SILCAAT. HsCRP was measured by ELISA by both laboratories, using a NBTMII nephelometer, N Antiserum to Human CRP (Siemens Diagnostics) for the SMART study, and an R&D Systems ELISA assay for ESPRIT and SILCAAT. The D-dimer and hsCRP assays, while different, compared very well on duplicate samples.[6] Lower limits of detection for D-dimer and hsCRP were 0.01 and 0.16 μg/mL, respectively, for SMART, and 0.045 and 0.078 μg/mL for ESPRIT and SILCAAT. All samples were analyzed blinded to clinical information about the participants.

### Statistical Methods

#### Associations of baseline biomarker levels with SNA/death

In order to describe associations of individual biomarkers with SNA/death, we computed Kaplan-Meier estimates of the cumulative risk of SNA/death by quartiles of baseline biomarker levels, and estimated hazard ratios (HRs) per 2x higher baseline biomarker levels in separate Cox proportional hazards models. All biomarkers were analyzed on the log_2_ scale, because their distributions were skewed on the original scale. All proportional hazards models were adjusted for age (categories 18–40, 41–50 and 51+ years), sex, and study (ESPRIT, SILCAAT, SMART), because these covariates were independently associated with SNA/death in a joint biomarker model.

In order to investigate whether IL-6, D-dimer and hsCRP are independently associated with the risk of SNA/death, we started with a model that contained all three biomarkers, as well as age, sex, race, study indicator, baseline CD4 cell counts (in categories ≤500, 500–800, 801+ cells/μL), nadir CD4, and indicator variables for the use of protease inhibitors (PIs) and non-nucleoside reverse transcriptase inhibitors (NNRTIs) at baseline, and stepwise eliminated covariates that did not improve the AIC model fit [38]. The final model contained log_2_ IL-6, log_2_ D-dimer, age, sex, and the study indicator, each with p-values ≤0.05. Because in the general population BMI is associated with the risk of CVD and death, we also tested for an association between BMI and the risk of SNA/death by adding BMI to the final biomarker model. We did not include BMI in the formal backwards variable selection procedure, because we were missing BMI values for 52 participants.

The IL-6 & D-dimer score was determined as the log HR estimate for given IL-6 and D-dimer levels using the final proportional hazards model, which included the two log_2_-transformed biomarkers, age, sex and the study indicator. Therefore, the score is a linear combination of the log_2_ biomarkers, weighted by the estimated regression coefficients. We tested the proportional hazards assumption using the product of the score with log-transformed time.

As sensitivity analyses, we estimated associations of IL-6, D-dimer, and the IL-6 & D-dimer score with each of the components of SNA/death in proportional hazards models, again adjusted for age, sex and study indicator. We compared the associations of the IL-6 & D-dimer score with the risk of SNA/death across subgroups by age, sex, and study cohort by testing for the interaction effects between score and subgroup indicator. In order to assess whether the association between the IL-6 & D-dimer score and the risk of SNA/death might have been driven by the presence of subclinical disease at baseline, we performed two analyses. First, we estimated the associations with events during the first 3 years (censoring later follow-up) and with later events (by excluding participants with events prior to year 3), and compared the associations during the earlier versus later time periods by adding the time-updated indicator function of years 4–10 to the proportional hazards model. Second, we compared the associations of the IL-6 & D-dimer score with the risk of SNA/death in participants who had experienced non-fatal SNA events prior to enrollment to the associations between score and risk in participants without prior SNA events, by testing for an interaction effect between the prior event status and the score. The latter analysis was restricted to the SMART and ESPRIT participants, because the history of prior non-AIDS events was not collected in the SILCAAT study.

#### Correction for regression dilution to estimate the effect of reducing the “usual” biomarker levels

Associations between a person’s “usual” biomarker levels (within-person long-term average levels) with the risk of SNA/death are stronger than those of single, baseline biomarker measurements, due to measurement error and random within-person fluctuations in the biomarkers over time. To correct for this regression dilution, the reliability coefficients for IL-6, D-dimer, and the IL-6 & D-dimer score were estimated using biomarker data collected at baseline, 1 and 3 years for 235 participants on stable ART in the ESPRIT study [39, 40]. To estimate the reliability coefficients, variance components were estimated using longitudinal mixed models for the biomarkers and the score, adjusted for visit by treatment group, age and sex. Similar adjustments for regression dilution bias have been carried out in previous studies of IL-6 and D-dimer [14–16]. After adjusting for regression dilution bias, we estimated the potential effect of a therapy-induced, sustained reduction of 0%-40% in IL-6 and/or D-dimer levels on the risk of SNA/death using proportional hazards models, and summarized the estimates and pointwise 95% confidence limits in contour plots.

We estimated the mean change in CD4 cell counts through follow-up using a longitudinal mixed model with random intercept and fixed slope, adjusted for study indicator.

Analyses used SAS version 9.3 (SAS Institute, Cary, NC, USA) and R version 3.1 [41]. All p-values are two-sided.

## Results

### Baseline characteristics

Baseline characteristics of the 3766 participants are summarized in Table 1. The median age was 42 years and 21% were women. At study entry, median time since HIV diagnosis was 7.3 years and participants had used ART for a median of 4.9 years. All participants had plasma HIV RNA levels ≤ 500 copies/mL. The median CD4 cell count was 500 cells/μL, median nadir 181 cells/μL. Twenty-seven percent had a prior AIDS diagnosis. Median (IQR) for IL-6, D-dimer and hsCRP were 1.7 (1.1–2.7) pg/mL, 0.22 (0.15–0.35) μg/mL and 1.54 (0.67–3.60) μg/mL, respectively (Table 1). The three biomarkers were pairwise correlated; Spearman’s correlations between IL-6 and D-dimer, IL-6 and hsCRP, and D-dimer and hsCRP were 0.31, 0.46, and 0.24, respectively (p<0.001 each).

**Table 1:** Baseline characteristics ^a^.

	Median (IQR) or %				
Characteristic	Overall		SMART	ESPRIT	SILCAAT
	(n = 3766)		(n = 1748)	(n = 1446)	(n = 572)
Age (years)	42	(37, 49)	45	40	41
Female (%)	21.2		25.8	17.4	16.6
Race/Ethnicity (%)					
Black	16.4		24.9	8.6	10.1
White/Latino/Other	83.6		75.1	91.4	89.9
Location of Enrollment (%)					
Asia	3.4		0.7	8.0	-
Australia, Europe, Israel	46.9		37.0	52.3	63.1
North America	37.4		49.0	23.5	36.9
South America	11.8		12.8	15.3	-
Africa	0.6		0.5	0.9	-
HIV-related factors					
CD4 cell count (cells/mm^3^)	500	(365, 690)	654	452	205
Nadir CD4 (cells/mm^3^)	181	(70, 290)	230	176	57
Prior AIDS (%)	27.1		24.9	28.1	31.3
Years since HIV diagnosis	7.3	(4.0, 11.4)	8.4	6.0	6.3
Duration of ART use (years)	4.9	(2.5, 8,0)	6.0	4.2	3.9
Current use of a PI (%)	47.8		42.1	47.9	64.9
Current use of a NNRTI (%)	50.8		52.0	51.7	44.8
BMI (kg/m^2^)	24.1	(22.0, 26.6)	24.7	23.7	23.7
**Biomarkers**					
IL-6 (pg/mL)	1.7	(1.1, 2.7)	1.6	1.9	1.7
D-dimer (μg/mL)	0.22	(0.15, 0.35)	0.18	0.26	0.25
hsCRP (μg/mL)	1.54	(0.67, 3.60)	1.72	1.42	1.34

Abbreviations: ART = antiretroviral therapy, hsCRP = high-sensitivity C-reactive protein, IL-6 = Interleukin-6, IQR = interquartile range, NNRTI = non-nucleoside reverse transcriptase inhibitor, PI = protease inhibitor.

^a^ All 3766 participants were using suppressive ART at baseline (HIV RNA ≤ 500 cp/mL), and were randomized to continue their ART regimen.

### Associations of baseline biomarker levels with SNA/death

**Fig 1:**
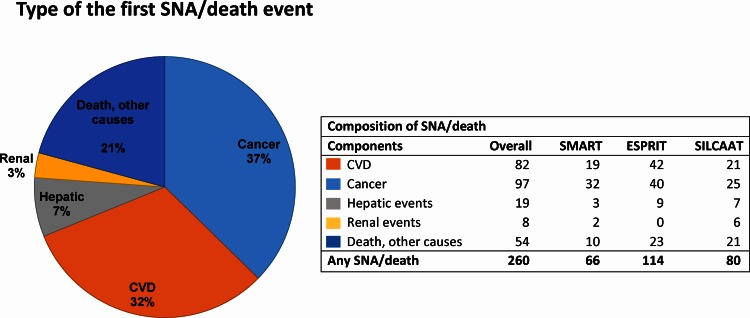
Composition of the serious non-AIDS disease or death (SNA/death) endpoint. Only the first event per participant is counted.

Over a mean follow-up of 4.9 years, 260 participants experienced SNA/death (1.44 per 100 person-years, 95% CI: 1.27 to 1.63). The first event was non-AIDS cancer for 97 participants (37%), CVD for 82 (32%; 45 myocardial infarction, 19 stroke, 18 deaths due to CVD), decompensated liver cirrhosis for 19 (7%), end-stage renal disease for 8 (3%), and death due to other causes for 54 (21%) participants, including 14 deaths due to AIDS, and 8 due to violence, accident or suicide (Fig 1).

**Fig 2:**
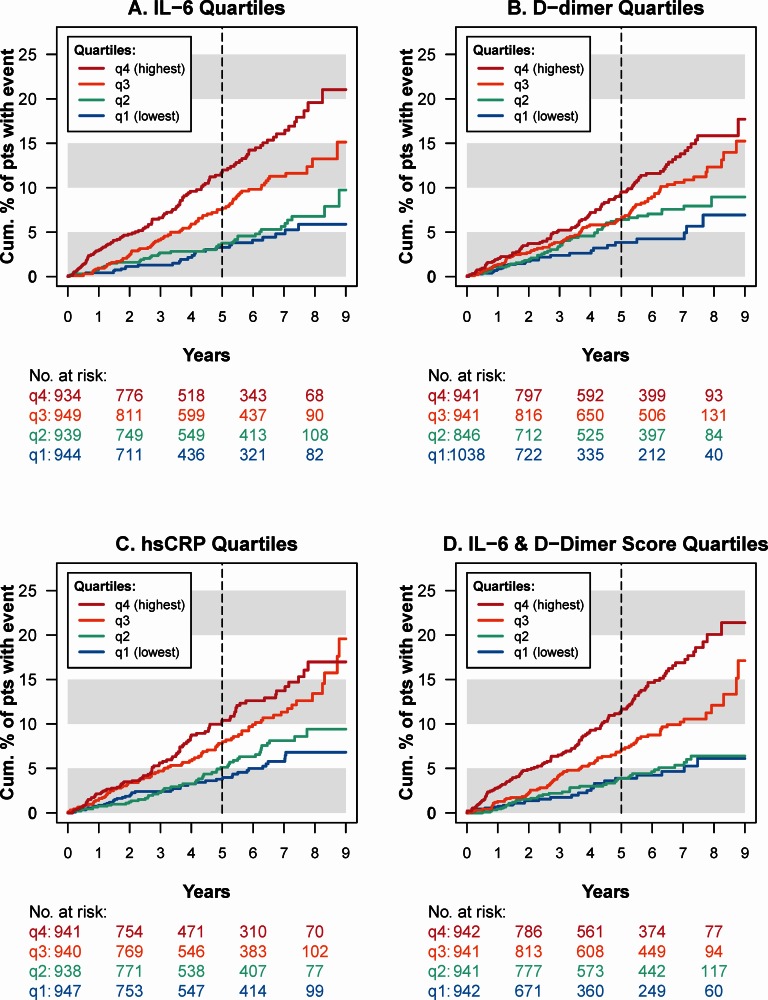
**Kaplan-Meier estimates of the cumulative proportion of participants who experienced serious non-AIDS disease or death (SNA/death), by quartiles of IL-6 (A), D-dimer (B), hsCRP (C), and the IL-6 & D-dimer score (D).** Biomarkers were measured at study entry. At 5 years, the risk of SNA/death was 12.0% for participants in the highest quartile of IL-6, compared with 3.5% for participants in the two lowest quartiles. Abbreviations: IL-6 = Interleukin-6; hsCRP = high-sensitivity C-reactive protein.

Fig 2, panels A-C, show Kaplan-Meier curves for time to SNA/death by quartiles of IL-6, D-dimer and hsCRP at study entry. High baseline levels of IL-6 and D-dimer predict an elevated risk of SNA/death over 5 years and longer; by year 5, the cumulative rate of SNA/death was 12.0% among participants in the highest quartile of IL-6, compared with 3.5% for participants in the lowest two quartiles (Fig 2A). The spread of the curves for D-dimer and hsCRP quartiles was less dramatic but still significant (Fig 2B and 2C).

**Fig 3:**
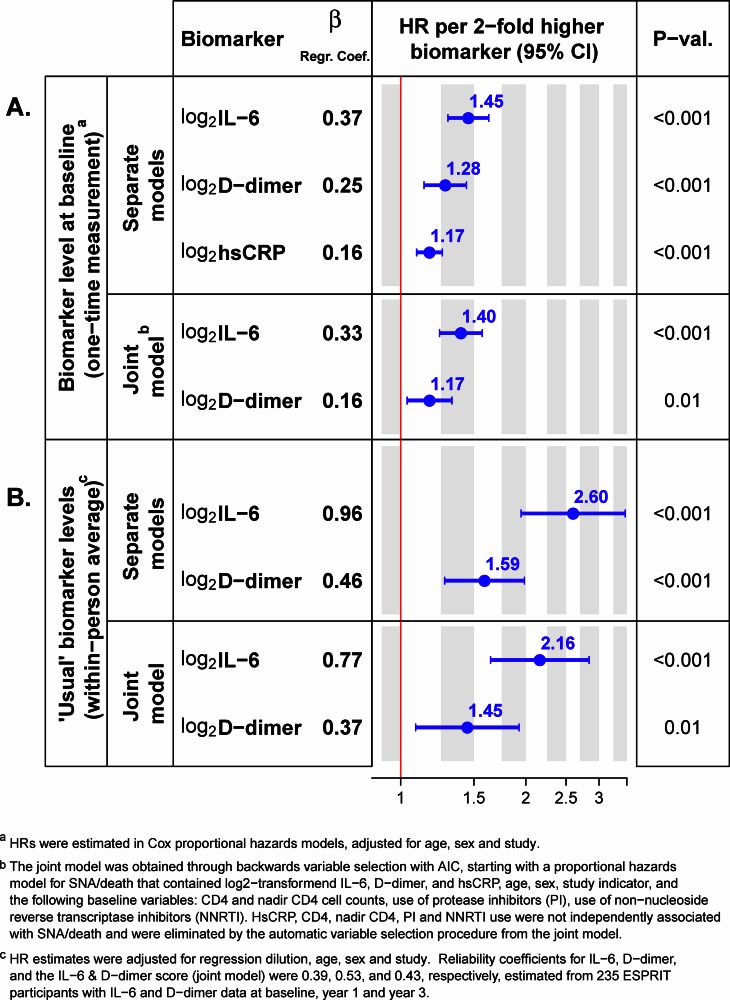
Hazard ratios (HRs) for serious non-AIDS conditions or death (SNA/death) per 2x higher levels of IL-6, D-dimer, and hsCRP. Panel A shows HRs for differences in baseline biomarker levels (a one-time measurement), estimated using proportional hazards models for each biomarker separately, and in a joint biomarker model containing both IL-6 and D-dimer. Panel B shows HRs for differences in “usual” (the within-subject long-term average) biomarker levels, estimated in separate and joint biomarker models after adjustment for regression dilution bias. All models were adjusted for age, sex, and study indicator (SMART, ESPRIT, SILCAAT). Abbreviations: IL-6 = Interleukin-6; hsCRP = high-sensitivity C-reactive protein.

Estimated hazard ratios of SNA/death per 2x higher baseline levels of IL-6, D-dimer and hsCRP are summarized in Fig 3A. In separate models, IL-6, D-dimer and hsCRP were each individually associated with risk of SNA/death; the estimated hazard ratios per 2x higher biomarker levels were 1.45 (95% CI: 1.30 to 1.63) for IL-6, 1.28 (95% CI: 1.14 to 1.44) for D-dimer, and 1.17 (95% CI: 1.09 to 1.26) for hsCRP; p<0.001 for each. All models were adjusted for age, sex and study.

Mean CD4 cell count levels increased slightly through follow-up, by 14 cells/mm^3^ per year.

### Development of the IL-6 & D-dimer biomarker score

We obtained a parsimonious joint biomarker model by applying backwards variable selection to a proportional hazards regression model containing all three biomarkers along with several other baseline factors. In the resulting *joint biomarker model*, both IL-6 and D-dimer were independently associated with the risk of SNA/death (Fig 3A, joint model), and the log hazard ratio for SNA/death per log_2_ IL-6 and log_2_ D-dimer units higher is estimated by the

$$\text{IL-}6\;\&\;\text{D-dimer score}:\;0.33\times \log_{2}\;\text{IL-}6+0.16\times \log_{2}\;\text{D-dimer}.$$

**Fig 4:**
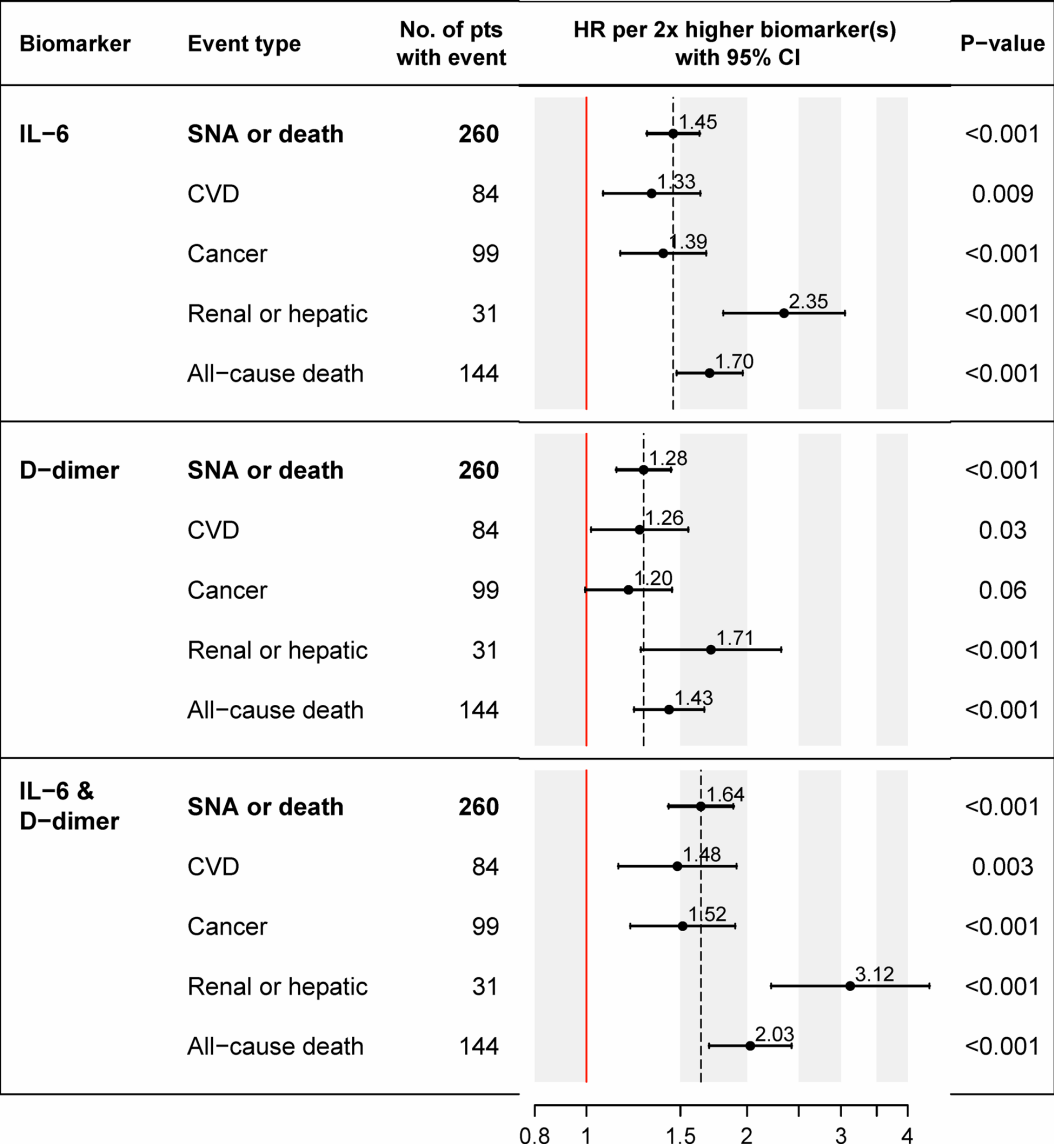
Estimated hazard ratios (HR) for the composite endpoint of serious non-AIDS or death (SNA/death) and its components. Hazard ratios are estimated per 2x higher level of IL-6 and D-dimer, with 95% confidence intervals. Two times higher biomarker levels corresponds to 1 unit higher on the log_2_ biomarker scale, and 0.49 units higher for the IL-6 & D-dimer score. Hazard ratios were estimated in proportional hazards models adjusted for age, sex and study. ***Footnotes*:** Of the 144 deaths, 37 were due to non-AIDS cancer, 33 CVD, 11 non-cancer hepatic disease, 1 renal failure, 15 AIDS-related, 9 due to violence, accident or suicide, 38 due to other or unknown causes. Abbreviations: CI = confidence interval; CVD = cardiovascular disease (myocardial infarction, stroke, and death due to CVD); HR = hazard ratio.

Higher levels of the IL-6 & D-dimer score were associated with increased risk of SNA/death (Figs 2D and 4) and each of its components (Fig 4). In particular, 2x higher levels of both IL-6 and D-dimer correspond to 1 unit higher for each of the two log_2_ biomarker components of the score, an IL-6 & D-dimer score of 0.49, and an estimated hazard ratio of 1.64 (95% CI: 1.43 to 1.89; p<0.001) for SNA/death.

In the joint biomarker model, the estimated risk of SNA/death was higher for older participants (HR = 2.0 for ages 41–50 versus 18–40 years, 3.3 for older than 50 years versus 18–40 years, both p<0.001), men compared with women (HR = 1.62, p = 0.01), and for participants in the SILCAAT study, which enrolled subjects with lower CD4 T cell counts (Table 1). The associations of the IL-6 & D-dimer score with the risk of SNA/death, however, were homogeneous across age, sex, or study (p >0.99, 0.94, and 0.70, respectively, for the interactions between the score and the factors). Baseline hsCRP, CD4, CD4 nadir, and use of PIs or NNRTIs at baseline did not contribute independently to the risk and were excluded by the variable selection procedure. Similarly, BMI was not independently associated with the risk of SNA/death in the joint biomarker model (p = 0.08).

Associations of the IL-6 & D-dimer score with the risk of SNA/death remained stable over time (p = 0.45 for a test of proportional hazards using log time). During the first 3 years, 2x higher baseline levels of both IL-6 and D-dimer were associated with a hazard ratio of 1.65 (95% CI: 1.37 to 2.00; p<0.001), while the hazard ratio was 1.61 (95% CI: 1.31 to 1.98; p<0.001) after the first 3 years (p = 0.69 for a difference in hazard ratios by time period). Associations were also consistent across the SMART, ESPRIT and SILCAAT studies (p = 0.86 for interaction between the IL-6 & D-dimer score and study indicator), across age groups (p = 0.95), and for men compared with women (p = 0.89).

Non-fatal non-AIDS events prior to study entry were reported in the SMART and ESPRIT studies. When excluding the 146 (4.6%) participants who had a prior myocardial infarction (35), stroke (28), non-AIDS cancer (42), liver cirrhosis or hepatic steatosis (42), or end-stage kidney disease (5), the hazard ratio for SNA/death among SMART and ESPRIT participants changed from 1.62 to 1.57 (95% CI: 1.31 to 1.87, p<0.001) per 2x higher baseline levels of IL-6 and D-dimer; associations between the IL-6 and D-dimer score and the risk of SNA events were similar for those with and without a history of prior SNA (p = 0.48 for the interaction).

#### Predicting the effect of reducing “usual” levels of IL-6 and/or D-dimer through therapy

Estimated reliability coefficients were 0.39 (95% CI: 0.28 to 0.50) for IL-6, 0.53 (95% CI: 0.44 to 0.62) for D-dimer, and 0.43 (95% CI: 0.33 to 0.54) for the IL-6 & D-dimer score. Associations of “usual” (within-person long-term average) biomarker levels with the risk of SNA/death are summarized in Fig 3B; in the joint biomarker model, the estimated hazard ratio per 2 times higher “usual” biomarker levels was 2.16 (95% CI: 1.64 to 2.84) for IL-6, 1.45 (95% CI: 1.09 to 1.93) for D-dimer, and 3.16 (95% CI: 2.28 to 4.37) for both. These hazard ratios were estimated in proportional hazards models, adjusted for regression dilution, and for age, sex and study.

**Fig 5:**
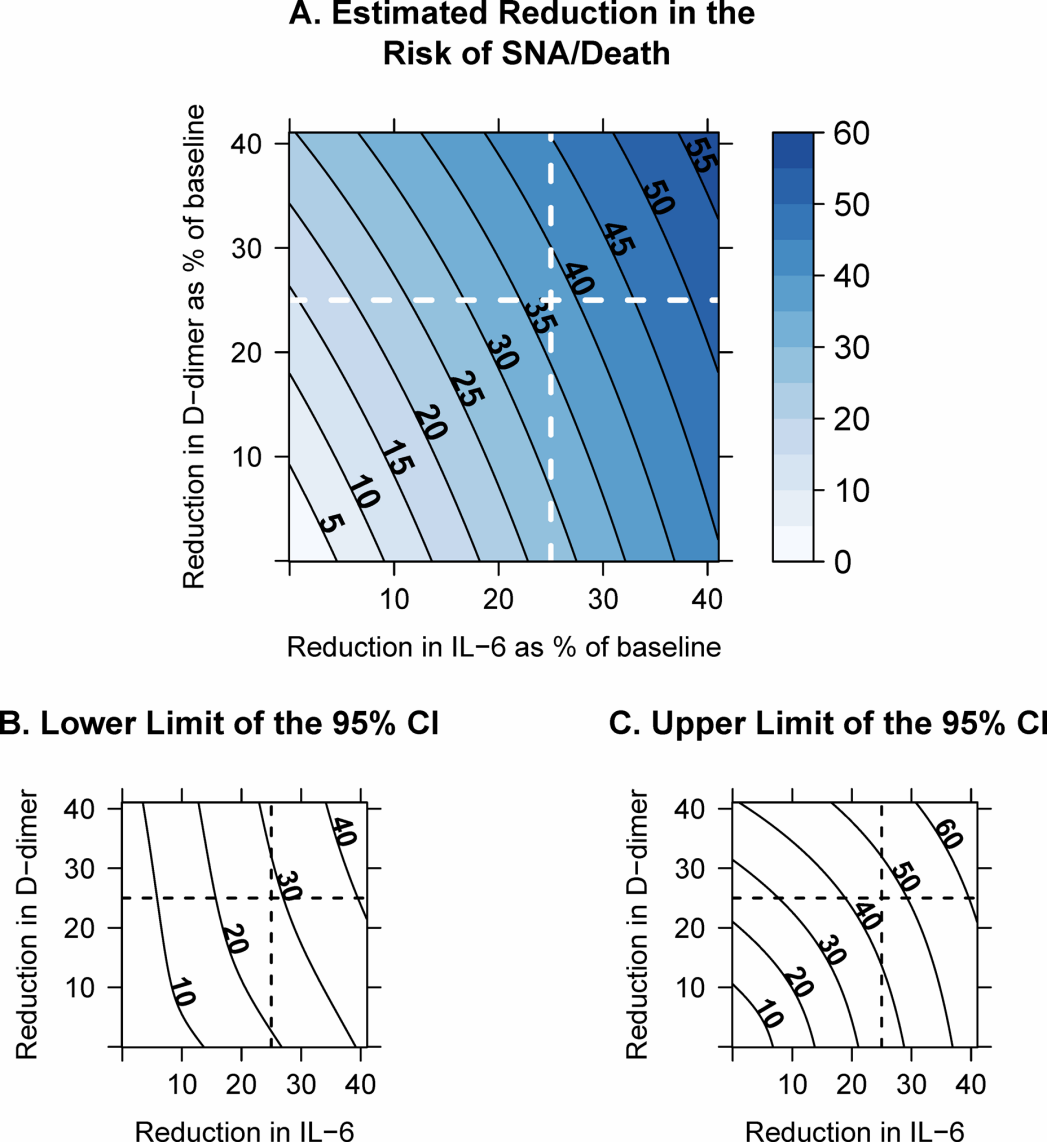
Estimated risk reduction. Panel A: Contour plot of the estimated reduction in the risk of serious non-AIDS conditions or death (SNA/death) when “usual” (within-person long-term average) levels of IL-6 and/or D-dimer are decreased by 0–40%, possibly through an intervention. Solid lines show predicted risk reductions of 5%, 10%, etc. Panels B and C show the lower and upper 95% confidence limits, respectively. For a 25% reduction in IL-6 and D-dimer, the risk of SNA/death is predicted to decline by 37% (95% CI: 28 to 46%). ***Footnotes*:** The reduction in the risk was estimated using Cox proportional hazards models, adjusted for regression dilution bias, and for age, sex, and study. Reliability coefficients for the regression dilution adjustment were estimated using longitudinal mixed models for biomarker data collected at baseline, years 1 and 3 for 235 participants on stable ART in the ESPRIT study. Abbreviations: CI = confidence interval; IL-6 = Interleukin-6.

Based on the joint biomarker model, after adjustment for regression dilution, we estimate that a sustained, long-term reduction in levels of IL-6 and D-dimer by 25% would result in a 37% reduction in the risk of SNA/death (95% CI: 28% to 46%) (Fig 5). The contour plot in Fig 5A shows the estimated reduction in the risk of SNA/death for combinations of different amounts (0%-40%) of sustained reductions in the “usual” IL-6 and/or D-dimer levels. A similar risk reduction by 37% would also be predicted for a sustained reduction in IL-6 by 30% and D-dimer by 12%, for example, or for a reduction in IL-6 by 20% and D-dimer by 33%. Limits of 95% confidence intervals are provided in Fig 5B and 5C.

## Discussion

Clinical endpoint trials are needed to establish whether lowering levels of inflammation and/or coagulation among HIV-positive individuals will decrease the risk of serious non-AIDS conditions, the leading cause of severe morbidity and mortality in virally well-controlled HIV infection. [1–4] We have developed a novel IL-6 & D-dimer biomarker score, which could be used as a primary biomarker outcome in phase II trials aiming to compare side-by-side drugs with different mechanisms of action (decrease of inflammation and/or coagulation), by evaluating treatments with respect to their model-predicted potential to reduce SNA/death.

We have shown that a single measurement of IL-6, D-dimer or hsCRP at baseline predicted the risk of SNA/death over 5 years and longer in a large cohort of HIV-positive individuals on suppressive ART regimens with moderate or high CD4 cell counts. Higher baseline levels of IL-6, D-dimer and hsCRP were associated with increased risk of SNA/death when considered separately, and IL-6 and D-dimer were independently associated with the risk in a joint biomarker model.

We combined CVD, cancer, serious renal and hepatic disease, and all-cause mortality into the composite outcome of SNA/death, because each of these conditions had been linked individually to high levels of inflammation and/or coagulation in the general population as well as among HIV-positive individuals. Markers of inflammation and coagulation have been most widely studied for their association with CVD [6, 7, 14–22] and all-cause mortality [10, 11, 27–29]. Currently, a clinical trial is ongoing to test whether reduction in inflammation through low-dose methotrexate reduces CVD or death in the general population [42], as well as a trial to test whether statin therapy prevents CVD among HIV-infected individuals using ART [43]. However, systemic inflammation and activation of coagulation appear to have broader effects beyond CVD. Elevated levels of IL-6, hsCRP and/or D-dimer were predictive of cancer, in particular death due to cancer [16, 23, 24, 44]; high D-dimer levels were linked to cancer mortality more strongly than to CVD [28]. Higher levels of inflammation and coagulation are also predictive of kidney function decline [26], and liver disease has been linked to increased coagulation [45]. In our cohort, associations of elevated IL-6 & D-dimer scores with the risk of cancer, kidney and liver disease, and all-cause mortality individually were at least as strong as associations with CVD. Associations of the IL-6 & D-dimer score with the risk of SNA/death were also consistent across studies (SMART, ESPRIT, and SILCAAT), across age groups, similar for men and women, and across baseline ART regimens.

Our biomarker models suggest that lowering levels of inflammation and/or coagulation might substantially decrease non-AIDS morbidity and mortality among HIV-positive patients on suppressive ART regimens, if inflammation and coagulation are on the causal pathway. Suitable agents to test this hypothesis in a clinical trial could target inflammation or coagulation to varying degrees, but would have to be safe for long-term use in combination with ART. Phase II trials would be needed to evaluate safety along with the potential to decrease inflammation or coagulation. The IL-6 & D-dimer score combines markers of inflammation and coagulation optimally to estimate the risk of SNA/death, and such could be used as biomarker outcome in pilot trials to compare several candidate agents.

Within the limitations of a prospective cohort study, we performed analyses to address the possibility that the associations of elevated baseline levels of IL-6 and D-dimer with the risk of SNA/death might have been due to subclinical disease which increased the biomarker levels as well as the subsequent risk of clinical disease. In our cohort, the strength of the associations between baseline biomarkers and risk of SNA/death remained stable through follow-up in, and associations of the IL-6 & D-dimer score with SNA/death were similar for participants with and without a prior history of non-fatal SNA events. These findings suggest that the associations are not explained by subclinical disease.

The main strength of our study was the large cohort of well-characterized participants, centrally determined biomarkers, the standardized definition of SNA events across the cohort, and the rigorous adjudication of the events in the SMART and ESPRIT cohorts by an independent ERC. The study has several limitations. First, we considered only three biomarkers; other markers might provide even stronger risk predictions. Second, the biomarkers were determined in two different central laboratories. Analyses of 20 repeated samples across the laboratories showed similar values, however [6]; also, all models included the study indicator, and thereby adjusted for potential differences between the two laboratories. Third, biomarker levels are highly variable within subjects, both due to assay variability as well as due to natural fluctuations. While we adjusted our estimates for the resulting regression dilution bias, the statistical uncertainty inherent in the biomarker measures resulted in large confidence intervals for the hazard ratios. Lastly, the SMART, ESPRIT and SILCAAT trials were conducted in 1997–2008, and it is possible that results would be slightly different in a population receiving more current ART regimens.

In summary, both IL-6 and D-dimer independently predict the risk of serious non-AIDS conditions or death among HIV-positive patients with suppressed virus and moderate to high CD4 cell counts. Treatments to decrease IL-6 and D-dimer might substantially reduce morbidity and mortality in HIV-positive patients on suppressive ART, since non-AIDS conditions dominate morbidity in this population. Clinical trials are needed to test this hypothesis. The IL-6 & D-dimer score could be a suitable biomarker outcome in phase II trials to compare drugs with different mechanisms of action (targeting inflammation, coagulation, or both) for their estimated, model-predicted potential to reduce non-AIDS morbidity and mortality.

## Supporting Information

S1 File Abbreviations. Abbreviations in alphabetical order.
(DOCX)
