# Supplementary material for: Relevance of Interleukin-6 and D-Dimer for Serious Non-AIDS Morbidity and Death among HIV-Positive Adults on Suppressive Antiretroviral Therapy
Source: PLoS One. 2016 May 12;11(5):e0155100. doi: 10.1371/journal.pone.0155100 (PMC4865234; doi:10.1371/journal.pone.0155100)
Supplement: S1 File — Abbreviations in alphabetical order. (DOCX) [file pone.0155100.s001.docx]

# Abbreviations

| ART | Antiretroviral therapy |
| --- | --- |
| CI | Confidence interval |
| CVD | Cardiovascular disease |
| ERC | Endpoint Review Committee |
| ESPRIT | Evaluation of Subcutaneous Proleukin® in a Randomized International Trial |
| hsCRP | High-sensitivity C-reactive protein |
| HR | Hazard ratio |
| IL-6 | Interleukin-6 |
| INSIGHT | International Network for Strategic Initiatives in Global HIV Trials |
| IQR | Interquartile range |
| NNRTI | Non-nucleoside reverse transcriptase inhibitor |
| PI | Protease inhibitor |
| SILCAAT | Subcutaneous Recombinant, Human Interleukin-2 in HIV-infected Patients with Low CD4+ Cell Counts under Active Antiretroviral Therapy |
| SMART | Strategies for Management of AntiRetroviral Therapy |
| SNA  SNA/death | Serious non-AIDS conditions  Serious non-AIDS conditions or death due to any cause |
|  |  |
